# Computational design of 3C-like protease substrate peptide for modular detection of protease activity of coronavirus

**DOI:** 10.48130/els-0026-0002

**Published:** 2026-06-22

**Authors:** Yuqi Han, Wei Guo, Long Sun, Siyu Du, Feifei Li, Tao Wang, Cheng Zhu

**Affiliations:** Tianjin Key Laboratory of Function and Application of Biological Macromolecular Structures, School of Life Sciences, Tianjin University, Tianjin 300072, China

**Keywords:** Protein design, Biosensor, Virus, Protease

## Abstract

The severity of the recent Coronavirus Disease 2019 pandemic stresses the importance of analytical and biosensor research aimed at determining and curbing disease severity. It demanded an easy-to-adapt method to reflect the infectivity of viruses. The critical role of 3C-Like protease (3CL^Pro^) in the replication cycle of coronaviruses, such as SARS-CoV-2, highlights its potential as an effective target correlated with viral activities. In this study, we aimed to develop a modular and orthogonal analytical tool for the detection and quantification of the essential protease component of coronaviruses, focusing on the enzymatic activity of the viral 3CL^Pro^, a key component for coronavirus replication. Our approach leveraged the high sequence conservation of 3CL^Pro^ across global coronavirus strains, particularly in its substrate recognition pocket, to computationally design optimal substrate peptides. The designed sequences were orthogonal to any natural viral protein sequences. When incorporated into Gluc or FlipGFP proteins, our designs achieved modular gain-of-signal or loss-of-signal detections of 3CL^Pro^ expression levels. These antigen-focused molecular tools could facilitate the screening of effective treatments and quantitative visualization of virus-infected cells.

## Introduction

Coronaviruses have been the etiological agents in several major pandemic outbreaks over the last two decades, including the Severe Acute Respiratory Syndrome (SARS), Middle East Respiratory Syndrome (MERS), and Coronavirus Disease 2019 (COVID-19)^[1−3]^. Together, these viral infections caused millions of yearly infections around the globe, with an estimated case fatality rate of 9.6% (SARS), 35% (MERS), and 1%~5% (COVID-19). With the development of oral inhibitors (e.g., Paxlovid) against COVID-19 and the constant viral evolution towards less lethal strains, the impact of COVID-19 illness on public health has been alleviated^[4,5]^. From a retrospective point of view, however, a significant number of casualties during the pandemic were due to the overwhelming of health care systems, due to the lack of means to effectively distinguish false-positive cases from severe infections^[6]^. Specifically, the well-established Real-Time Quantitative PCR (RT-qPCR), as the standard method to monitor viral agents in a large population, may not demonstrate the differences between live viruses and debris of dead viruses^[7]^. Hence, the design of orthogonal analytical tools for detection and quantification of the conserved component of functional coronaviruses would be desirable for tracking more viral variants and providing hierarchical treatments in the future.

The common molecular diagnostic methods for various types of viruses could be categorized as nucleic acid-based, antibody-based, or antigen-based^[8]^. Among the nucleic acid amplification tests, the polymerase chain reaction (PCR) assays were recognized as the gold standard. The isothermal amplification (e.g., LAMP) and CRISPR-based tests were later developed to simplify the procedure^[9]^. Nonetheless, viral RNA may circulate in the patient's body for ~90 d, even after the virus loses infectivity, making it difficult to determine the stage of infection. The serologic test of antibodies (e.g., ELISA) was not primarily used to diagnose current infections, because the specific antibodies were produced after the immune system reacted to, and almost cleared up the viruses. To increase the sensitivity of immunoassays, a combined IgM/IgG antibody test was thus derived^[10,11]^. The antigen detection assays usually focus on the E, N, and S proteins of the Severe Acute Respiratory Syndrome Coronavirus 2 (SARS-CoV-2)^[12]^. While the viral surface glycoprotein S underwent constant mutations to escape the humoral immune system, the nucleocapsid protein N shares ~90% and ~49% sequence identity with that of SARS and MERS-CoV, respectively, making the N protein a preferred target for detection. However, these assays heavily relied on antibodies that recognized epitopes on N or S; thus, the read-outs reflected 'existence' instead of viral 'activity'.

To overcome this barrier, we hypothesized that an indispensable component for coronavirus replication, the 3C-Like protease (3CL^Pro^, or main protease M^Pro^), could become a useful indicator of viral infectivity^[13,14]^. Unlike the N protein and S protein, the enzymatic feature of 3CL^Pro^ could facilitate a direct readout of the 'activity signal'. By incorporating its substrate into a modular construct of luciferases or green fluorescent proteins (GFP), the activity of 3CL^Pro^ or the infectivity of SARS-CoV-2 could correlate with light signals. More importantly, by rationally optimizing the substrate octapeptide of 3CL^Pro^, it is possible to increase 3CL^Pro^-recognition specificity and the detection sensitivity. We set out to test this hypothesis by computationally designing substrates of SARS-CoV-2 3CL^Pro^ with elevated binding energies and assembling the molecular and cellular detection systems as a proof-of-concept for the potential detection of live viruses.

## Results

### Structure-based design of 3CL^Pro^-binding sequences

**Figure 1:**
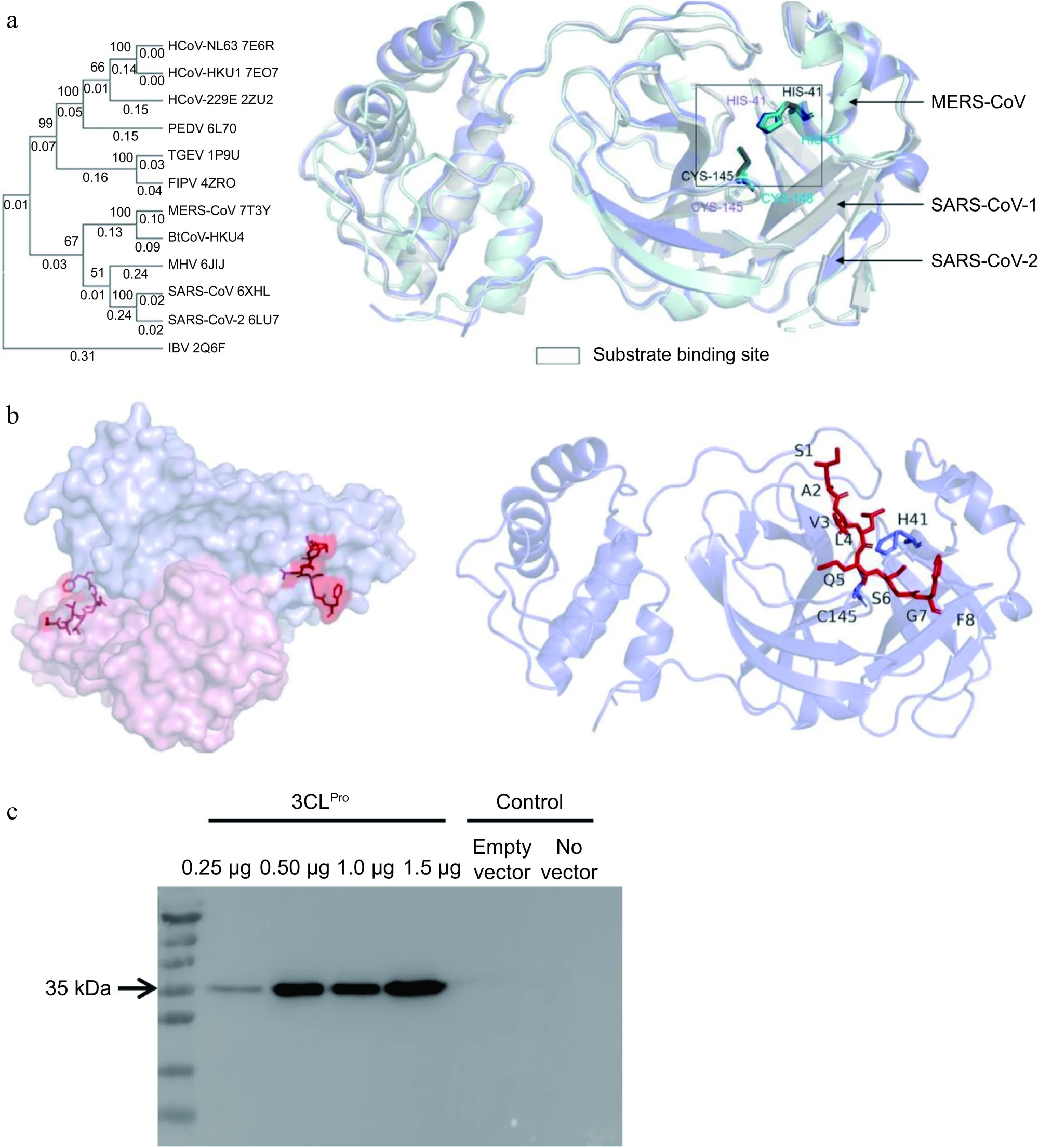
The substrate binding pocket of 3CL^Pro^. (a) Structural alignment of three coronaviruses 3CL^Pro^ (SARS-CoV-1, SARS-CoV-2, and MERS-CoV). The catalytic dyad was indicated in the black box. (b) The substrate peptide (red sticks) bound to 3CL^Pro^. The protease was a homodimer in native conformations. In the design process, only one protomer (right panel) was used as the input model. (c) 3CL^Pro^ expression in HEK-293T cells. The cells were transfected with different amounts of pCMV vectors encoding 3CL^Pro^ (33.8 kD, without the Flag tag) and the protein expression levels were detected by Western blot using anti-Flag antibodies.

Throughout the life cycle of coronaviruses, 3CL^Pro^ performed the protease function after viral mRNA translation, but not before the assembly of nucleocapsid proteins into particles. It cleaved 12 sites within the polyprotein precursors pp1a and pp1ab^[15]^. Hence, the enzymatic activity of 3CL^Pro^ was directly linked to replication and maturation of coronaviruses. 3CL^Pro^ possesses > 96% sequence identity among global coronavirus strains (Supplementary Figs S1, S2). The most conserved region of 3CL^Pro^ was the substrate recognition pocket and the catalytic dyad (H41 and C145), (Fig. 1a)^[4,13]^. Thus, we hypothesized that a linear peptide that closely fits into the 3CL^Pro^ pocket would resist the majority of drifting mutations in virus evolution and be a starting point for deriving a 3CL^Pro^ detection tool.

The catalytic pocket of SARS-CoV-2 3CL^Pro^ was a shallow groove accommodating a curved peptide conformation (Fig. 1b), composed of key interacting residues T24, N142, H163, E166, and Q189, with H41 and C145 flanking the cleavage site (Leu-Gln↓ [Ser, Ala, and Gly]). Based on the crystal structure of 3CL^Pro^ and the native peptide (SAVLQSGF, PDB 7N89)^[16]^, we applied the deep learning sequence design model ProteinMPNN to optimize the peptide sequence while keeping the cleavage site intact^[17]^. To diversify the peptide sequences, we also applied Rosetta FastDesign to find optimal combinations of the residue rotamers with flexible backbones^[18,19]^. In total, we obtained three sequences from Rosetta FastDesign and one sequence from ProteinMPNN (out of 1,000 ranked designs) (Table 1). We predicted the complex structural models of 3CL^Pro^ and designed peptides using AlphaFold3, and the results indicated that all peptides bound at the pocket, adopting similar conformations as compared to the native models. To indicate the orthogonality and specificity of the P1 sequence, we performed a BLAST search for homologies and found that the closest protein segment was from the phosphohydroxythreonine transaminase of Bacillota bacterium. Hence, it is distinct from any eukaryotic proteins.

**Table 1:** Computational characterization of WT and designed sequences.

ID	Sequence	AlphaFold3 predictions	Rosetta energy (REU)
WT	SAVLQSGF	ipTM = 0.8	−56.1
P1	PVILQYTT	ipTM = 0.72	−75.8
P2	MSRLQTSN	ipTM = 0.73	−64.3
P3	PVILNYTH	ipTM = 0.64	−71.5
P4	KPRLQAGN	ipTM = 0.69	−47.5

To mimic the viral infections and set up a model system for examination of our biosensor designs, we expressed 3CL^Pro^ in the HEK-293T cell line (Fig. 1c) by plasmid transfections. We demonstrated that a varying concentration of 3CL^Pro^ could be achieved by varying the concentration of plasmids, thus semi-quantitatively mimicking the amounts of virus that infected the cell. We adopted this transcription system in the following experiments.

### Modular design of luciferase and GFP-based 3CL^Pro^ detection tools

#### IN-GLuc designs

**Figure 2:**
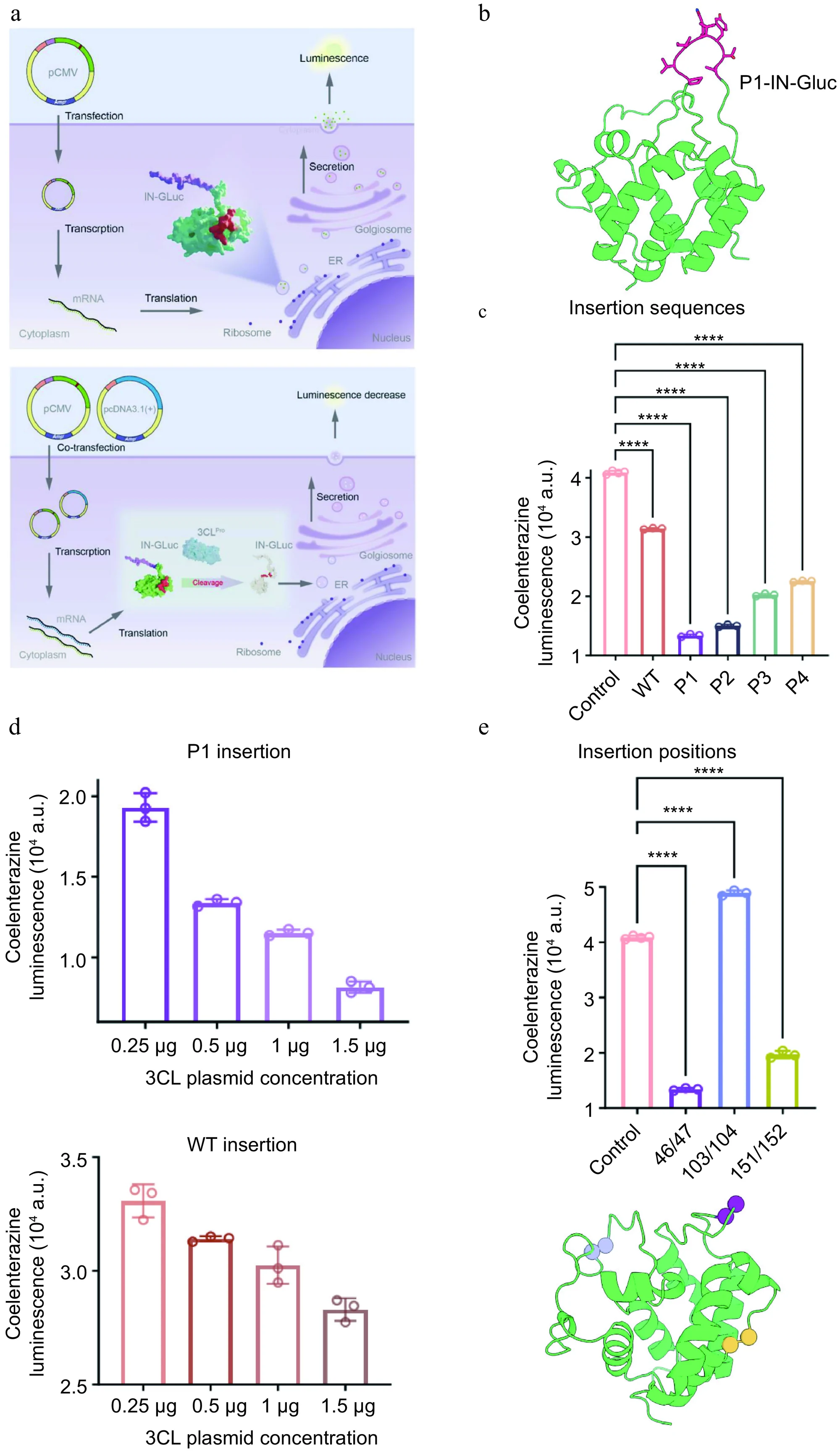
The design of IN-Gluc. (a) The design principle of IN-Gluc. 3CL^Pro^ cleaved the peptide sequence inserted in the middle of Gluc, a secreted enzyme catalyzing the oxidation of coelenterazine to a luminescent molecule, leading to a reduction of luminescence (lower panel). In the absence of 3CL^Pro^ or infections, the Gluc was readily secreted out of the cytosol (upper panel). (b) The AlphaFold3 predicted structural model of P1-IN-Gluc. (c) The effects of different designed sequences when inserted into Gluc. The control was deactivated 3CL^Pro^ H41A/C145A expressed at the same level. (d) The 3CL^Pro^ dose-dependent signal change when P1 or WT peptide was inserted into Gluc. (e) The effects of different insertion positions and the locations of 46/47, 103/104, and 151/152 positions indicated as spheres. *n* = 3 as the number of replications (*n* = 4 for the control sample).

We first considered Gaussia Luciferase (GLuc, ~19.9 kD secreted enzyme), one of the brightest naturally occurring luminescent enzymes, as the scaffold for insertion of 3CL^Pro^-recognition peptides^[20−22]^. When 3CL^Pro^ was present and active, the peptide-inserted Gluc proteins would result in a reduction of bioluminescent signals (Fig. 2a). Two factors were optimized when constructing the peptide-insertion proteins (denoted as IN-Gluc): the insertion sites and inserted sequence. We used SPELL, an online prediction tool for the identification of proper insertion sites on the surface of GLuc, avoiding disruption of the overall protein structure^[23]^. SPELL suggested the residue pairs 46/47, 103/104, and 151/152 for incorporating the designed sequences (the structural model in Fig. 2e). We first compared the effects of the native substrate (WT: SAVLQSGF) and all designed substrates when they were inserted into 46/47 (Fig. 2b, c). When 3CL^Pro^ was expressed in the cell, the WT-IN-Gluc featured a 23% reduction of Gluc signals, while P1-IN-Gluc featured a 67% reduction of signals (the highest among designed sequences, Fig. 2c), indicating that a tight binding between the peptide and 3CL^Pro^ would facilitate more thorough cleavage.

We then induced different expression levels of 3CL^Pro^ by transfecting various concentrations of vectors. The signals from P1-IN-Gluc semi-quantitatively corresponded to the amounts of 3CL^Pro^ with a dynamic range between 52% and 80%, wider than that of WT-IN-Gluc (18% to 30%). Hence, we focused on the P1 design in the following studies.

The 103/104 insertion did not result in signal reduction; by contrast, the luminescence increased 18% when 3CL^Pro^ was expressed (Fig. 2e). We think it is possible that the two halves of Gluc that split at the 103/104 site are associated with each other and function as a whole, releasing conformational tensions from the inserted P1 sequence. The 151/152 insertion resulted in 52% signal reduction. We also tested a combination of two different insertions, such that both 46/47 and 181/182 were inserted with P1 to increase the chance of recognition by 3CL^Pro^. However, the double-insertion designs probably interfered with the catalytic function of Gluc, and only 35%~47% signal responses were recorded, less than the 67% reached by a single insertion at 46/47. Hence, a balance between 3CL^Pro^ recognition and the structural integrity of Gluc should be considered in these designs. In conclusion, P1-IN-Gluc was the best among the similar designs.

#### TM-Gluc designs

**Figure 3:**
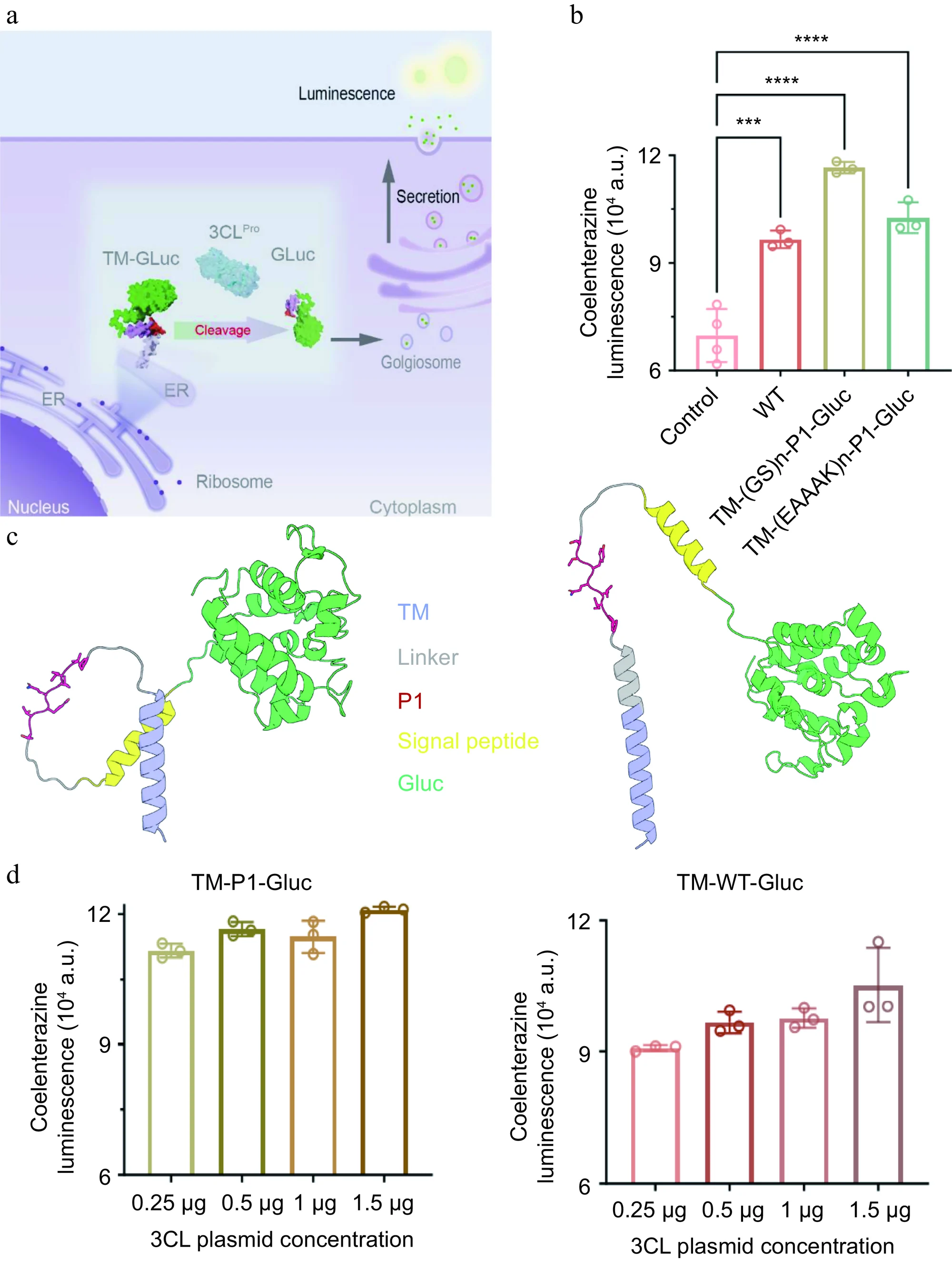
The design of TM-Gluc. (a) The design principle of TM-Gluc. 3CL^Pro^-mediated cleavage released Gluc for extracellular secretion, leading to elevation of luminescence. (b) The effects of flexible linker and rigid linker when fusing the P1 peptide with the membrane anchoring sequence TM. (c) The AlphaFold3 predicted structural model of P1-TM-Gluc (left: flexible linker, right: rigid linker). (d) The 3CL^Pro^ dose-dependent signal change when P1 or WT peptide was fused at the N-terminal of Gluc. *n* = 3 as the number of replications (*n* = 4 for the control sample).

Instead of inducing a drop of light signal, we took advantage of the secretion property of Gluc and installed a transmembrane helix (TM) at the terminus of Gluc to retain it in the cytosol (Fig. 3a)^[24]^. We then put the P1 peptide between TM and Gluc (Fig. 3c). The active 3CL^Pro^ would release Gluc out of the cytosol so that we could measure the signals in the extracellular medium that reflected the amount of secreted Gluc. We consistently adopted the P1 sequence while optimizing one factor in the TM-Gluc design: the linker sequence (flexible or rigid).

We found that a flexible linker (GS)_n_ combined with N-terminus TM-P1-Gluc could result in a 47% signal increase. A rigid linker (EAAAK)_n_ resulted in less sensitive changes of signal (37% increase). The TM-(GS)_n_-P1-Gluc also quantitatively responded to the changing levels of 3CL^Pro^ with a dynamic range of 42% to 55%. Compared to the IN-P1-Gluc design, TM-(GS)_n_-P1-Gluc featured higher basal levels of fluorescein luminescence (0.8 × 10^7^ to 1.5 × 10^7^) at comparable Gluc expression levels, thus making it easier for detection. We postulated that the reason for this difference was that inserting P1 into the Gluc might interrupt its enzymatic activity, while fusing P1 at the N-terminus of Gluc minimizes this structural interruption. Compared to TM-(EAAK)_n_-P1-Gluc with the rigid linker, the sensitivity of TM-(GS)_n_-P1-Gluc was higher for the same amount of 3CL^Pro^ expression. Hence, for gain-of-signal detections, the TM-(GS)_n_-P1-Gluc was a suitable module.

#### IN-FlipGFP designs

**Figure 4:**
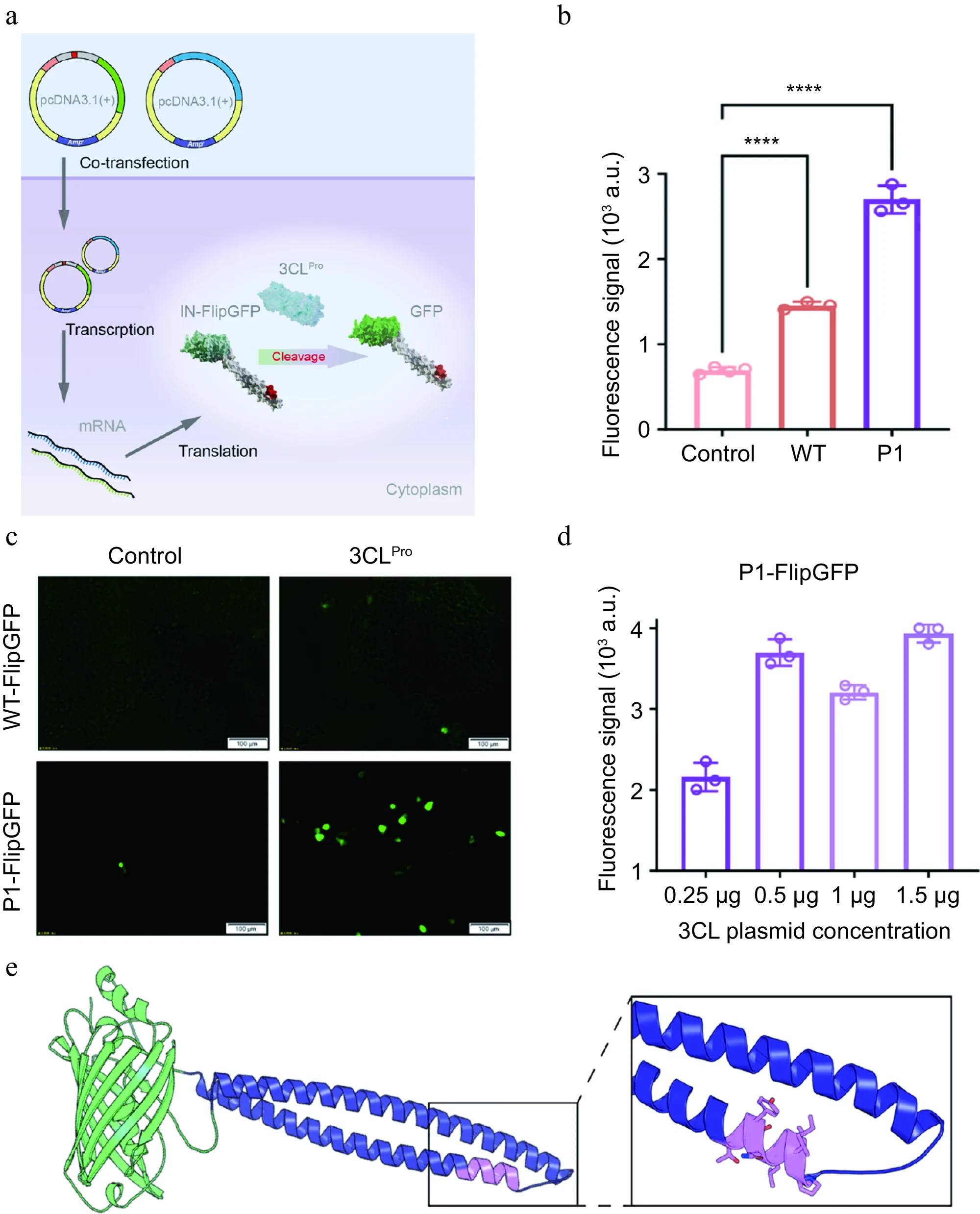
The design of IN-FlipGFP. (a) The design principle of IN-FlipGFP. 3CL^Pro^-mediated cleavage released conformational constraint of FlipGFP and restored the green fluorescence. (b) The comparison between WT peptide insertion and P1 peptide insertion in the FlipGFP construct. (c) The image of cells expressing 3CL^Pro^ and IN-FlipGFP indicating the brightest signal came from P1-IN-FlipGFP, with 3CL^Pro^ H41A/C145A expression as the control (left panel). (d) The 3CL^Pro^ dose-dependent signal change of the P1-IN-FlipGFP construct. (e) The AlphaFold3 predicted structural model of P1-IN-FlipGFP with the insertion site highlighted. *n* = 3 as the number of replications (*n* = 4 for the control sample).

As the second method to assemble 'signal elevation' systems, we inserted the P1 peptide into the previously reported FlipGFP^[25,26]^. The intrinsic fluorescence of enhanced Green Fluorescent Protein (eGFP) was abolished by flipping two β-strands with tightly bound coiled-coil helices (Fig. 4a). We inserted P1 and WT peptides into the coiled-coil region of FlipGFP, respectively (the structural model in Fig. 4e). Without 3CL^Pro^ expression, the cell cultures stay in a dark state. 3CL^Pro^ cleavage of the substrates restored FlipGFP to the light state, with the green fluorescence increasing 3.9 times for P1-IN-FlipGFP and 1.2 times for WT-IN-FlipGFP (Fig. 4b). This system is more sensitive than TM-P1-Gluc, spanning a 2.0- to 5.0-fold fluorescence elevation when expression of 3CL^Pro^ increased 6 times (Fig. 4c, d).

### Application of designed 3CL^Pro^ sensor

3CL^Pro^ was one of the major drug screening targets for the treatment of coronaviruses. Nirmatrelvir (USA), Ensitrelvir (Japan), and Leritrelvir (China) were 3CL^Pro^-specific inhibitors prescribed in post-exposure prophylaxis^[27]^. We envisioned that molecular tools that detect the activity of 3CL^Pro^ would facilitate drug screening. As proof of principle, we used P1-IN-Gluc as the reporter, as the drug inhibition would lead to a signal increase. In the 3CL^Pro^ active cell line, the signals from P1-IN-Gluc remain at basal levels (7.5 × 10^4^). When 50 nM and 100 nM Paxlovid were added to the cell culture, we observed dose-dependent elevation of luciferase signals at 1.5 × 10^5^ and 3.0 × 10^5^ (Supplementary Fig. S3), respectively, suggesting a quantitative 3CL^Pro^ activity reporter was useful to demonstrate drug efficacy.

## Methods

### Computational design of 3CL^Pro^ substrate sequence

The crystal structure of 3CL^Pro^ and the native substrate SAVLQSGF (PDB 7N89) complex was relaxed by Rosetta and used as the input for substrate sequence design. The FastDesign mover of Rosetta was applied to find the optimal combinations of residues at each position (eight positions in total) in Cartesian mode. The backbones and side chains were flexible during the design process, allowing bond angles and bond lengths to be altered and sampled. The 3CL^Pro^ proteins were allowed to relax when the peptide sequence was altered. The canonical amino acids were sampled for the peptide except for cysteine (P1 in Table 1). The binding energy was evaluated by the Rosetta energy function with RigidBody TransMover. The design and evaluation scripts were based on PyRosetta4.Release. python312 and provided in the Supplementary Material.

To diversify the designed sequences, an enhanced sampling of the hydrogen bond network between the peptide and 3CL^Pro^ was also tested using HBNetStapleInterface movers. More polar residues were placed in the peptide by this mover (P3 and P4 in Table 1).

The deep neural network model ProteinMPNN was also applied to design the peptide. Five thousand sequences were designed with a sampling temperature of 0.1 and the vanilla_model_weights v_48_020. The designs were ranked by global score, and the top-ranking sequences were selected for experimental evaluations (P2 in Table 1).

### Plasmid constructs

The gene encoding 3CL^Pro^ was synthesized by Tsingke Biotech (Beijing, China) and cloned into the pcDNA3.1 (+) vector. The codons were optimized for mammalian cell expression. As a control construct, the inactive version of 3CL^Pro^ was constructed by mutating H41 and C145 (active site) to alanine (H41A/C145A). The Gaussia luciferase gene sequence was recorded on www.snapgene.com/plasmids/luciferase_vectors/pCMV-Gaussia_Luc and cloned into the pCMV vector for mammalian cell expression. The eGFP and FlipGFP genes were also obtained from SnapGene and cloned into pcDNA3.1(+) vectors (www.snapgene.com/plasmids/fluorescent_protein_genes_and_plasmids/EGFP). The insertion of P1~P4 peptide sequences into luciferase or FlipGFP was conducted by TansGen Biotech PCR Kit with TransStart® FastPfu DNA Polymerase (Beijing, China) and a two-step overlapping PCR approach. The synthesized and purified plasmids were sequenced using fluorophore-based Sanger sequencing. The corresponding protein sequences were recorded in Supplementary Table S1.

### Cell cultures

The human embryonic kidney cells (HEK-293T, ATCC #CRL-3216) were cultured in Dulbecco's Modified Eagle Medium (DMEM) basic (Gibco, Cat#C11995500BT) containing 10% fetal bovine serum (FBS) (Gibco, Cat#10270-106), 100 μg/mL streptomycin, and 1% penicillin (MeilunBio, #MA0110). The cell cultures were typically grown on a 12-well plate at a 37 °C incubator with 5% CO_2_. At the 60%–80% confluency, the plasmids encoding 3CL^Pro^ or 3CL^Pro^ H41A/C145A (200–1,500 ng), IN-Gluc/TM-Gluc/IN-FlipGFP (100 ng) were transfected using Lipofectamine 3000 (Thermo Scientific, #L3000015). For the measurement of inhibitor effects, nirmatrelvir (PF-07321332) was purchased from MedChemExpress (99.83% purity) and applied to cell cultures at 50 or 100 nM end-point concentrations for 30 min before signal readout.

### Measurement of Gluc bioluminescent signals

After 24 h of transfection, the luciferin bioluminescent signals were detected on a Centro microplate luminometer Centro XS^3^ LB 960 (Dixie Scientific), using Pierce™ Gaussia Luciferase Glow Assay Kit (Thermo). Typically, 15 μL of cell culture media was collected, and 50 μL of Gaussia Glow Assay Buffer containing coelenterazine was added for 10 min of incubation before the reading.

### Measurement of eGFP signals

After 24 h of transfection, cells were fixed with 2% paraformaldehyde at room temperature for 15 min, then washed with PBS three times. The green fluorescence signals were observed on an Olympus IX71 Inverted Fluorescence Microscope (excitation at 489 nm and emission at 511 nm). The signal strength was quantified by ImageJ.

### Statistical analysis

Quantification was performed using GraphPad Prism v8.0.2. Statistical significance was determined using Student's t-test to compare experimental and control groups and indicated as *p* values (*p* < 0.05 as significant). The error bars indicated standard deviations.

## Discussion

Computational protein design could be categorized as energy function-based ('classical') and deep neural network-based ('AI') methods. These methods have been applied to either design protein biosensors from scratch or optimize existing molecular tools. The AI-based methods, including ProteinMPNN and LigandMPNN, were advantageous in the *de novo* design of novel proteins^[17,28,29]^. In comparison, the classical design methods, through redesigning binding partners or fusing functional domains, were more suitable for creating biosensors through the assembly of protein fragments. Notably, a modular and tunable protein pair, lucCage/lucKey, was designed with Rosetta by splitting luciferase and fusing the two fragments into helical bundles. This protein biosensor switched conformations (between bound and unbound states) to detect the presence of antigens like SARS-CoV-2 RBD and Botulinum neurotoxin B. However, the lucCage/lucKey was not designed to reflect enzymatic activities (e.g., protease activities). In our study, we took the 'sequence optimization' to mean optimizing the peptide substrate of a viral protease, 3CL^Pro^. This designed sequence (P1) became the foundation of new experimentally tested IN-Gluc, TM-Gluc, and IN-FlipGFP sensors. Based on the high-resolution crystal structures and the chemical environment of its catalytic pocket, the Rosetta protein design package was able to find optimal residues of the protease substrate and increase the sensitivity of these sensors, in comparison to the sensors equipped with a natural substrate (WT in Figs 2–4). We also compared the performance of Rosetta and ProteinMPNN (P1 and P2 in Table 1) and found that the classical design method performed better in this design task, possibly due to the limited length of the 3CL^Pro^ substrate (small search space) and explicit consideration of binding energies during the sequence design. Ideally, a more comprehensive screening and a broader search of designed sequences could lead to a peptide with suitable protease reaction kinetics (k_cat_/K_M_). The current work, although combining computational design and a quick screen with single read-outs (Fig. 2), may be limited in searching the vast peptide space.

An array of recent research was devoted to the development of 3CL^Pro^-focused assays during the COVID-19 pandemic. These cell-based reporter assays demonstrated different aspects of advantages, including performing at BioSafety Level 2 lab settings, being fast and accurate, having elevated S/N ratios, or enabling high-throughput compatible protocols^[20−22,24−26,30]^. By focusing on the optimization of the cleavable peptide, we demonstrated the flexibility of various protein biosensors in terms of readily integrating a new P1 sequence into different detection tools. In drug screening campaigns, these assays accounted for membrane permeability and bioavailability in cells, compared to those of *in vitro* assays (e.g., RT-qPCR). The limitation of this work and our study is that we used single-target detection, while the coronavirus possessed both 3CL^Pro^ and PL^Pro^ (Papain-like protease) as indispensable proteases^[31,32]^. A combined detection of dual targets may further eliminate false positivity in the future, which could be achieved by using our computational design protocol (Supplementary Text 1, Supplementary Fig. S4) and replacing the substrate peptide among Gluc or FlipGFP modules, as demonstrated in this study. Another limitation of our work is the detection limit and dynamic response range of these platforms. We propose that the sensitivity of any Gluc or FlipGFP-based platform could be enhanced by using P1 as the substrate. To test the possible detection limit, we reduced the amount of 3CL^Pro^ expression vectors to 20 ng and detected the minimal fluorescence signals (Fig. 4c). In the future, a more rigorous relationship between live virus infection (pfu/mL) and 3CL^Pro^ expression levels (mg/mL) should be established for an accurate limit of detection value. In summary, these tools not only offer a complementary approach to existing nucleic acid and antibody-based diagnostics but also hold promise for revolutionizing the way we monitor and control a wide range of pathogens for public health.

## Conclusions

In this study, we developed a modular and orthogonal biosensing strategy for detecting coronavirus activity by targeting the enzymatic function of 3CL^Pro^. Through structure-guided computational design, we identified an optimized substrate peptide (P1) with enhanced binding affinity and specificity toward 3CL^Pro^. Incorporation of this designed substrate into multiple reporter systems, including IN-Gluc, TM-Gluc, and IN-FlipGFP, enabled both loss-of-signal and gain-of-signal detection modalities with improved sensitivity compared to native substrates.

These biosensors demonstrated the ability to semi-quantitatively reflect 3CL^Pro^ expression levels in living cells and were further validated for evaluating the efficacy of protease inhibitors. By directly linking signal output to viral protease activity, this approach provides a functional readout of viral infectivity rather than mere presence.

Overall, our work establishes a versatile platform for activity-based viral detection and drug screening and highlights the potential of combining computational protein design with modular biosensor engineering for rapid response to emerging pathogens.

## SUPPLEMENTARY DATA

Supplementary data to this article can be found online.

## Data Availability

All data supporting the findings of this manuscript are available from the corresponding author (Wang T and Zhu C) upon reasonable request.
