# Supplementary material for: Computational design of 3C-like protease substrate peptide for modular detection of protease activity of coronavirus
Source: Eng Life Sci. 2026 Jun 22;26(5-6):e002. doi: 10.48130/els-0026-0002 (PMC13599429; doi:10.48130/els-0026-0002)
Supplement: Supplementary file 1 [file els-2026-26-5-6-0002-S1.zip › 10.48130_els-0026-0002-Suppl-FigureS2.pdf]

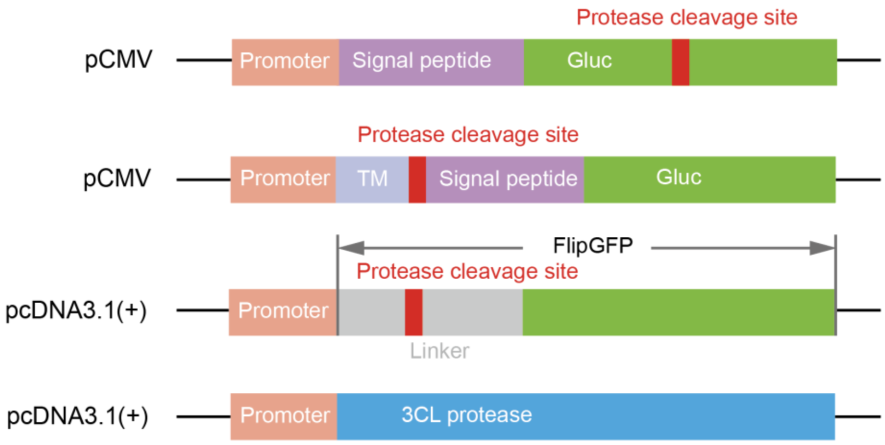

Figure S2. Schematics of plasmids construct used in this study.
