# Supplementary material for: Computational design of 3C-like protease substrate peptide for modular detection of protease activity of coronavirus
Source: Eng Life Sci. 2026 Jun 22;26(5-6):e002. doi: 10.48130/els-0026-0002 (PMC13599429; doi:10.48130/els-0026-0002)
Supplement: Supplementary file 1 [file els-2026-26-5-6-0002-S1.zip › 10.48130_els-0026-0002-Suppl-FigureS4.pdf]

A

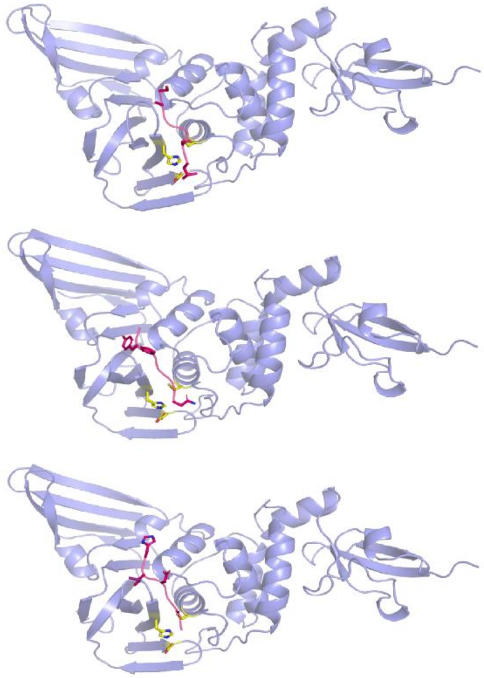

B

ID	sequence	AlphaFold3 predictions	Rosetta Energy (REU)
**WT**	LXGG↓XX	0.67	-43.1
**Rosetta Design**	GYKGGN	0.70	-53.1
**ProteinMPNN Design**	HIDGAG	0.54	-54.6

Figure S4. Structural models of PLpro (papain-like protease) and its peptide substrate. The catalytic triad was shown as yellow sticks. (A) WT peptide (shown as red linear strand, upper panel), Rosetta designed peptide (middle panel), ProteinMPNN designed peptide (lower panel). (B) The calculated binding capacity of PLpro peptide substrate.
