# Supplementary material for: Computational design of 3C-like protease substrate peptide for modular detection of protease activity of coronavirus
Source: Eng Life Sci. 2026 Jun 22;26(5-6):e002. doi: 10.48130/els-0026-0002 (PMC13599429; doi:10.48130/els-0026-0002)
Supplement: Supplementary file 1 [file els-2026-26-5-6-0002-S1.zip › 10.48130_els-0026-0002-Suppl-TableS1.pdf]

Table S1. The protein sequences used in this study.

Proteins	Sequences and Vectors
3CL^{pro}	SGFRKMAFPSGKVEGCMVQVTCGTTTLNGLWLDDVVYCPRHVICTSEDMLNPNYEDLLIRKSN<br>HNFLVQAGNVQLRVIGHSMQNCVLKLKVDTANPKTPKYKFVRIQPGQTFSVLACYNGSPSGVYQ<br>CAMRPNFTIKGSFLNGSCGSVGFNIDYDCVSFCYMHHMELPTGVHAGTDLEGNFYGPFVDRQTA<br>QAAGTDTTITVNVLAWLYAAVINGDRWFLNRFTTTLNDFNLVAMKYNYEPLTQDHVDILGPLSA<br>QTGIAVLDMCASLKELLQNGMNGRTILGSALLEDEFTPFDVVRQCSGVTFQ、 pcDNA3.1 (+)
3CL^{pro} H41A/C145A	SGFRKMAFPSGKVEGCMVQVTCGTTTLNGLWLDDVVYCPRAVICTSEDMLNPNYEDLLIRKSN<br>HNFLVQAGNVQLRVIGHSMQNCVLKLKVDTANPKTPKYKFVRIQPGQTFSVLACYNGSPSGVYQ<br>CAMRPNFTIKGSFLNGSAGSVGFNIDYDCVSFCYMHHMELPTGVHAGTDLEGNFYGPFVDRQTA<br>QAAGTDTTITVNVLAWLYAAVINGDRWFLNRFTTTLNDFNLVAMKYNYEPLTQDHVDILGPLSA<br>QTGIAVLDMCASLKELLQNGMNGRTILGSALLEDEFTPFDVVRQCSGVTFQ、 pcDNA3.1 (+)
GLuc	MGVKVLFALICIAVAEAKPTENNEDFNIVAVASNFATTDLDADRGKLPGKKLPLEVLKEMEANAR<br>KAGCTRGCLICLSHIKCTPKMKKFIPGRCHTYEGDKESAQGGIGEAIVDIPEIPGFKDLEPMEQFIA<br>QVDLCVDCTTGCLKGLANVQCSDLLKKWLPQRCATFASKIQGQVDKIKGAGGD、pCMV
WT-IN-Gluc	MGVKVLFALICIAVAEAKPTENNEDFNIVAVASNFATTDLDADRGSAVLQSGFKLPGKKLPLEVLK<br>EMEANARKAGCTRGCLICLSHIKCTPKMKKFIPGRCHTYEGDKESAQGGIGEAIVDIPEIPGFKDL<br>EPMEQFIAQVDLCVDCTTGCLKGLANVQCSDLLKKWLPQRCATFASKIQGQVDKIKGAGGD、<br>pCMV
P1-IN-Gluc	MGVKVLFALICIAVAEAKPTENNEDFNIVAVASNFATTDLDADRGPVILQYTTKLPGKKLPLEVLK<br>EMEANARKAGCTRGCLICLSHIKCTPKMKKFIPGRCHTYEGDKESAQGGIGEAIVDIPEIPGFKDL<br>EPMEQFIAQVDLCVDCTTGCLKGLANVQCSDLLKKWLPQRCATFASKIQGQVDKIKGAGGD、<br>pCMV
TM-(GS)n-P1-Gluc	VPVLVMSLLFIASVFMLHIWGSGSGSPVILQYTTGSGSGSMGVKVLFALICIAVAEAKPTENNEDF<br>NIVAVASNFATTDLDADRGKLPGKKLPLEVLKEMEANARKAGCTRGCLICLSHIKCTPKMKKFIP<br>GRCHTYEGDKESAQGGIGEAIVDIPEIPGFKDLEPMEQFIAQVDLCVDCTTGCLKGLANVQCSDL<br>LKKWLPQRCATFASKIQGQVDKIKGAGGD、pCMV
TM-(EAAK)n-P1-Gluc	VPVLVMSLLFIASVFMLHIWEAAAKEAAAKPVILQYTTGSGSGSMGVKVLFALICIAVAEAKPTE<br>NNEDFNIVAVASNFATTDLDADRGKLPGKKLPLEVLKEMEANARKAGCTRGCLICLSHIKCTPKM<br>KKFIPGRCHTYEGDKESAQGGIGEAIVDIPEIPGFKDLEPMEQFIAQVDLCVDCTTGCLKGLANVQ<br>CSDLLKKWLPQRCATFASKIQGQVDKIKGAGGD、pCMV
eGFP	MVSKGEELFTGVVPILVELDGDVNGHKFSVSGEGEGDATYGKLTLKFICTTGKLPVPWPTLVTTL<br>TYGVQCFSRYPDHMKQHDFFKSAMPEGYVQERTIFFKDDGNYKTRAEVKFEGDTLVNRIELKGI<br>DFKEDGNILGHKLEYNYNSHNVYIMADKQKNGIKVNFKIRHNIEDGSVQLADHYQQNTPIGDGP<br>VLLPDNHYLSTQSALSKDPNEKRDHMVLLEFVTAAGITLGMDELYK、pcDNA3.1 (+)
WT-IN-FlipGFP	ATMDLPDDHYLSTQTILSKDLNSGLRSGSGLEMEVSALEKEVSALEKEVSALEKEVSALEKEVSA<br>LEKEKRDHMVLLEYVTAAGITDASAVLQSGFKVSALKEKVSALKEKVSALKEKVSALKEKVSAL<br>KEDIEGRGSLLTCGDVEENPGPKLATMRKGEELFTGIVPILVELDGDVNGHKFFVRGEGEGDATIG<br>KLSLKFICTTGKLPVPWPTLVTTLTYGVQCFSRYPDHMKRHDFFKSAMPEGYVQERTIYFKDDGT<br>YKTRAEVKFEGDTLVNRIELKGIDFKEDGNILGHKLEYNFNSHKVYITADKQNNGIKANFTIRHN<br>VEDGSVQLADHYQQNTPIGDGPVLLPGGRSR、pcDNA3.1 (+)
P1-IN-FlipGFP	ATMDLPDDHYLSTQTILSKDLNSGLRSGSGLEMEVSALEKEVSALEKEVSALEKEVSALEKEVSA<br>LEKEKRDHMVLLEYVTAAGITDASPVILQYTTKVSALKEKVSALKEKVSALKEKVSALKEKVSA<br>LKEDIEGRGSLLTCGDVEENPGPKLATMRKGEELFTGIVPILVELDGDVNGHKFFVRGEGEGDATI<br>GKLSLKFICTTGKLPVPWPTLVTTLTYGVQCFSRYPDHMKRHDFFKSAMPEGYVQERTIYFKDDG<br>TYKTRAEVKFEGDTLVNRIELKGIDFKEDGNILGHKLEYNFNSHKVYITADKQNNGIKANFTIRH<br>NVEDGSVQLADHYQQNTPIGDGPVLLPGGRSR、pcDNA3.1 (+)
