# Supplementary material for: Computational design of 3C-like protease substrate peptide for modular detection of protease activity of coronavirus
Source: Eng Life Sci. 2026 Jun 22;26(5-6):e002. doi: 10.48130/els-0026-0002 (PMC13599429; doi:10.48130/els-0026-0002)
Supplement: Supplementary file 1 [file els-2026-26-5-6-0002-S1.zip › 10.48130_els-0026-0002-Suppl-Text1.pdf]

Supplementary Text 1: Scripts for peptide sequence design.

1 Scripts for peptide sequence design

The peptide sequence was Chain C, residue ID 1~8 in the structural model.

```
from pyrosetta import *
from pyrosetta.rosetta import *

pyrosetta.init("-ignore_unrecognized_res 1 -ex1 -ex2aro")

start_pose = pose_from_pdb("3CL.pdb")
pose = start_pose.clone()
scorefxn = pyrosetta.create_score_function("ref2015_cart.wts")
#select design residues
select_res
pyrosetta.rosetta.core.select.residue_selector.ResidueIndexSelector("1C,2C,3C,6C,7C,8C")
not_design_res = pyrosetta.rosetta.core.select.residue_selector.NotResidueSelector(select_res)

tf = pyrosetta.rosetta.core.pack.task.TaskFactory()
```

```
tf.push_back(pyrosetta.rosetta.core.pack.task.operation.InitializeFromCommandline())
tf.push_back(pyrosetta.rosetta.core.pack.task.operation.IncludeCurrent())
tf.push_back(pyrosetta.rosetta.core.pack.task.operation.NoRepackDisulfides())

# Disable design (i.e. repack only) on not_res
tf.push_back(pyrosetta.rosetta.core.pack.task.operation.OperateOnResidueSubset(
    pyrosetta.rosetta.core.pack.task.operation.RestrictToRepackingRLT(), not_design_res))

# Enable design on select_res
aa_to_design = pyrosetta.rosetta.core.pack.task.operation.RestrictAbsentCanonicalAASRLT()
aa_to_design.aas_to_keep("ADEFGHIKLMNPQRSTVWY")
tf.push_back(pyrosetta.rosetta.core.pack.task.operation.OperateOnResidueSubset(
        aa_to_design, select_res))
packer_task = tf.create_task_and_apply_taskoperations(pose)

mmf = pyrosetta.rosetta.core.select.movemap.MoveMapFactory()
mmf.all_bb(setting=True)
mmf.all_bondangles(setting=True)
mmf.all_bondlengths(setting=True)
mmf.all_chi(setting=True)
mmf.set_cartesian(setting=True)

fr = pyrosetta.rosetta.protocols.relax.FastRelax(scorefxn_in=scorefxn, standard_repeats=2)
fr.set_task_factory(tf)
fr.set_movemap_factory(mmf)
fr.apply(pose)
```
